# The Link Between Frontotemporal Dementia and Autoimmunity: A Case Presentation and Literature Review

**DOI:** 10.7759/cureus.24617

**Published:** 2022-04-30

**Authors:** Krysten Corzo, Banu Farabi, Lokesh Lahoti

**Affiliations:** 1 Medical School, St. George’s University School of Medicine, True Blue, GRD; 2 Internal Medicine, Saint Peter’s University Hospital, New Brunswick, USA

**Keywords:** dementia, autoimmunity, antiphospholipid antibody syndrome, rheumatoid arthritis, frontotemporal dementia

## Abstract

Dementia disorders are an important public health issue and thus of particular clinical importance. Frontotemporal dementia, although less prevalent than Alzheimer’s disease, presents in a significant number of cases in younger populations. Yet, it is a comparatively rare disease process, with a low yearly incidence. Frontotemporal dementia remains an exciting and ever-evolving area of research with most recent studies investigating the role of inflammation in the degeneration pathognomonic of the disease. Here, we describe a case that highlights the connection between inflammation and neurodegeneration. Specifically, we examine a patient with long-standing rheumatoid arthritis and antiphospholipid syndrome who developed frontotemporal dementia, potentially as a result of the chronic inflammatory state.

## Introduction

Frontotemporal dementia, one of the many dementia disorders, is a group of syndromes secondary to progressive degeneration involving the frontal and temporal regions of the brain. This pathological process presents as three main variants, namely, behavioral, nonfluent-agrammatic primary progressive aphasia, and semantic primary progressive aphasia [1]. Research suggests that early in the disease process, inflammation plays a leading role. It is suggested that the brain, through genetic predisposing or otherwise, experiences misfolding of tau proteins, which serves as a nidus for an inflammatory response to begin. Though the exact process is currently being researched, the alteration of the extra and intracellular environment within the brain due to inflammation may be the cause of the neurodegeneration specifically seen in frontotemporal dementia [2]. Current treatment options are geared toward symptom control through therapies and behavioral modification strategies with minimal success in disease progression. Various pharmacological agents are often utilized to target specific symptoms; however, the evidence supporting these treatments is weak. Future treatment options will focus on the components of this neuroinflammation in an attempt to alter disease progression.

Rheumatoid arthritis is an autoimmune disease attacking the body’s own synovial membranes. A process mediated by B-cells, T-cells, and monocytes prompts synovial hyperplasia, pannus formation, and, ultimately, bone and cartilage breakdown. This chronic inflammatory state mediated by the body’s immune response clinically presents as “soft” swelling, pain, and stiffness in multiple symmetric joints throughout the body. Extra-articular symptoms may also present as subcutaneous nodules and vasculitis, among others. Treatment of the disease centers around preventing disease progression and controlling symptoms. Conventional synthetic disease-modifying anti-rheumatic drugs such as methotrexate, hydroxychloroquine, and sulfasalazine are first-line choices for long-term treatment. If conventional treatments are not sufficient, then biologic agents such as tocilizumab and infliximab can be utilized [3].

Antiphospholipid syndrome (APS) is an autoimmune disorder known for thromboses throughout the body. Although the exact pathogenesis remains an area of research, inflammation plays a key role in APS [4]. This disorder utilizes inflammatory and immune players such as anticardiolipin antibodies, anti-B2 glycoprotein I antibodies, lupus anticoagulants, and complement through C4 and C3b [4]. In doing so, the disorder’s effect is systemic, involving an array of manifestations ranging from cardiac to renal and to neuropsychiatric manifestations. Among the neuropsychiatric manifestations are well-characterized complications of cerebral ischemia and less understood non-thrombotic manifestations of cognitive dysfunction and dementia [5]. Conventionally, treatment options for clinical manifestations that are a consequence of thrombosis are warfarin anticoagulation and for non-thrombotic symptoms are antiplatelet therapy.

Here, we describe a case of a male patient diagnosed with frontotemporal dementia with key risk factors, rheumatoid arthritis, and APS.

## Case presentation

A 65-year-old man with a history of APS, rheumatoid arthritis, and frontotemporal dementia presented to the emergency room with a complaint of altered mental status. According to the patient’s wife who witnessed the event, the patient was staring out a window when he suddenly lost his footing and slumped over the back of a chair. When approached by his wife, the patient was seen to be drooling with tearing and rhinorrhea. The patient was able to visually track his wife but was unable to respond to her questions appropriately. At the time, the patient’s speech was incoherent and slurred compared to his baseline. The entire event lasted for 10 minutes when the patient returned to baseline. There was no facial droop, tongue biting, loss of bowel or bladder control, or syncope. There were no previous diagnoses of hypertension or diabetes mellitus.

The patient had experienced a chronic battle with rheumatoid arthritis. Diagnosed with the condition in his early 20s, he had tried multiple medication regimens including prednisone, methotrexate, and infliximab. He was on medication to control joint pain since then. Deformities as a result of rheumatoid arthritis left the patient with an effortful uneven gait. Additionally, he had undergone bilateral knee replacements and surgery in his left elbow secondary to changes from the autoimmune inflammatory disease. Currently on tofacitinib, the patient was able to ambulate with minimal assistance around his house with a slow unsteady gait. Although his level of interaction was limited due to his dementia, the patient complained of pain in multiple joints.

The patient had experienced a stroke 15 years ago which presented as a facial droop and left no residual deficits. Unfortunately, no further information about this incident was known via either the patient’s family members or the available medical records. Historically, the patient was suffering from a progressive dementia disorder for the past five years. Initially, the patient’s symptoms began as mild forgetfulness, such as forgetting acquaintances’ names, and a decline in his usual interest to socialize. Then the patient experienced a prolonged episode of grief following the passing of his mother. During this time, the patient developed a low mood which, as per his wife, never returned completely to his prior emotional baseline. However, the patient was never formally diagnosed with any psychiatric condition and did not receive any treatment. Shortly afterward, the patient stopped going to work, frequently became lost while driving in familiar areas, and needed assistance with instrumental activities of daily living. He gradually ceased bathing and grooming himself. The patient’s condition worsened two years ago. While the patient was able to recall distant events and distant skills from early adulthood, he had difficulty retaining any new tasks, such as using a cellular phone. He became fixed in repetitive restrictive routines and often became agitated when deviating from those routines. Further changes included only eating sweets and urinating in inappropriate places, as well as a progressive hypophonia to the point that the patient did not speak above a low slow whisper. At times, he would ramble on about topics inconsequential to the questions asked; symptomatology that had worsened in severity and frequency over the years. Most recently, the patient had begun excessively scratching himself. Currently, the patient was able to complete most activities of daily living with full assistance from his wife though he exhibited increased agitation with having to do so. A diagnosis of frontotemporal dementia was formally given by the patient’s neurologist three weeks before presentation at the hospital with use of supportive brain imaging and extensive neuropsychiatric testing.

**Figure 1:**
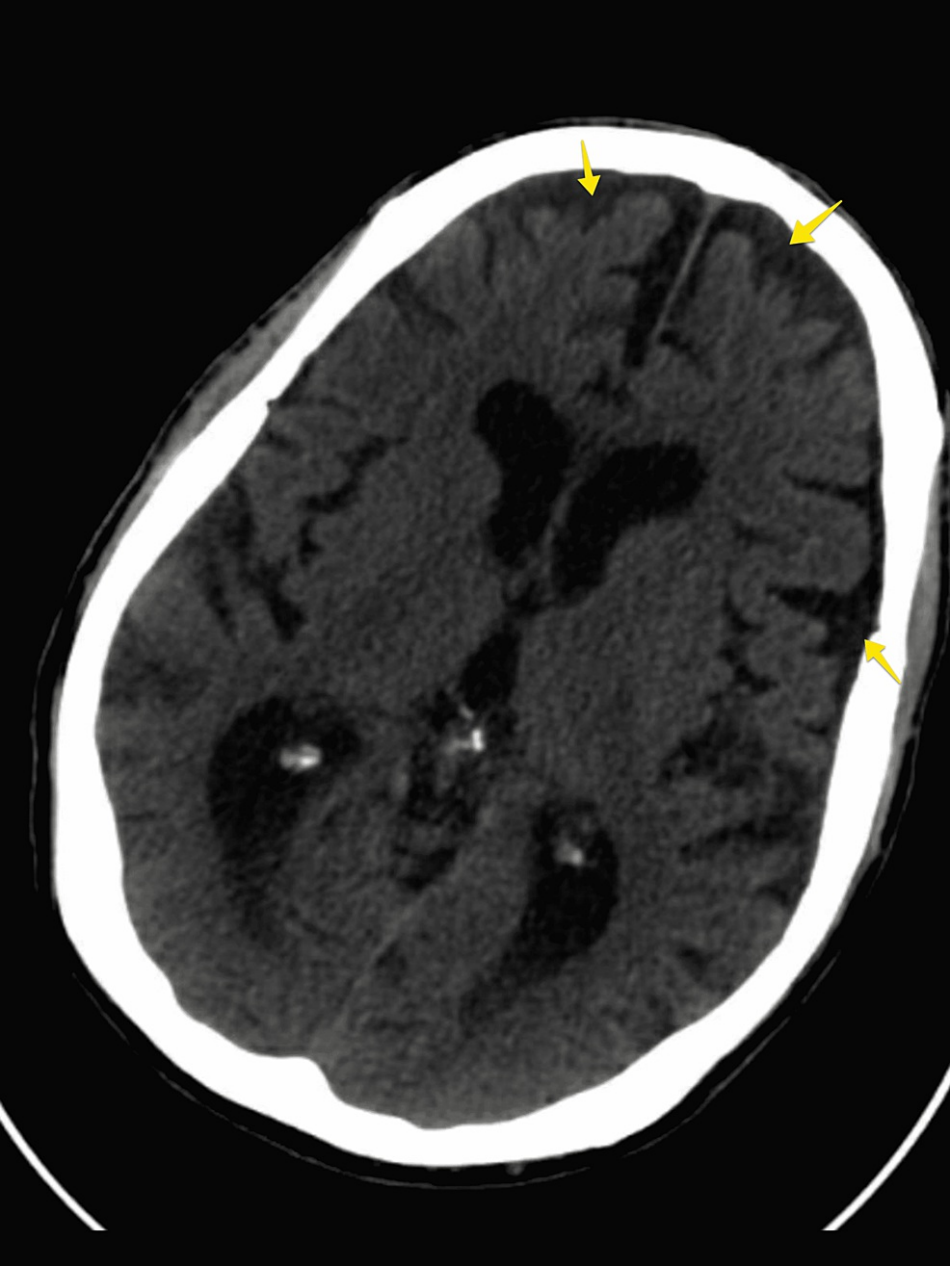
A non-contrast CT scan with arrows highlighting evident cerebral atrophy of the frontal and temporal regions. CT: computed tomography

On presentation to the emergency room, vital signs were within normal limits except for blood pressure of 166/91 mmHg. Pertinent positives on physical examination included the patient not being alert or oriented. Sparingly, the patient would respond to questions, doing so in a slow hypophonic voice. His range of motion was limited in the upper and lower extremities due to pain with movement. Cogwheel rigidity was noted in the left wrist. No joint swelling was noted at the time. Computed tomography of the head yielded evidence of moderate cerebral atrophy of the frontal and temporal regions (Figure 1). This was unchanged from three weeks ago. There were no new hemorrhages or infarcts noted. Investigative studies were limited due to the patient’s uncooperativeness. As such, magnetic resonance imaging could not be done. Although this presents as a potential limitation of the case, as an ischemic injury could not be ruled out via magnetic resonance imaging, the patient’s presentation was not similar to his previous stroke and was thought to be more likely related to progressive neurodegenerative presentations. Given that the patient had returned to baseline and other potential causes for his episode were ruled out through laboratory exams and electrocardiogram, it was further determined that the ataxic episode represented another symptom of his progressive disease process. The patient was discharged to follow up with his neurologist.

## Discussion

Observationally, the correlation between proinflammatory states and dementia disorders has been noted for some time [6]. One retrospective study analyzing data from 2001 to 2012 found that individuals with autoimmune rheumatic diseases were 1.18 times more likely to develop a dementia disorder than the general population [7]. Another retrospective study that examined data from 1980 to 2009 in Minnesota found that rheumatoid arthritis patients were 1.37 times more likely to develop a dementia disorder than those without the disease [8]. Similarly, about 2.5% of patients diagnosed with APS develop a dementia disorder [5]. In recent years, further information has emerged supporting a physiological link between chronic inflammation states, such as rheumatoid arthritis and APS, and the development of dementia disorders, including frontotemporal dementia [9]. One theory postulates that inflammation leads to reduced vascular supply to the brain. This leads to hypoxia and oxidative stress, as well as the decreased efflux of cellular waste and accumulation of malformed proteins. Another tandem theory is superficially described as the following: inflammation creates a disorganized cellular environment leading to entanglement of protein; these neurofibrillary tangles impair cellular organization and thus the movement of nutrients within the cells; such repeated cellular insults over time lead to atrophy; and this cellular damage manifests as the clinical presentation of dementia [9].

To further add insult to injury, some medical treatments for rheumatoid arthritis have been linked to the development of dementia. Such treatments include disease-modifying drugs such as methotrexate, sulfasalazine, and hydroxychloroquine [10]. For example, a recent multinational case-control study found that among rheumatoid arthritis patients, those taking methotrexate for over four years had a higher risk of developing dementia compared to those not using methotrexate [11].

Frontotemporal dementia has no cure as of today. As such, prevention, symptomatic control, and slowing progression are the techniques at our disposal. Being aware of chronic inflammatory states, such as rheumatoid arthritis and APS, as dementia risk factors is important to keep a patient’s quality of life as optimal as possible for as long as possible. Although lifetime inflammatory changes in the brain and throughout the body may seem inevitable according to current knowledge, we have come to find that some of these changes may be preventable [12]. Studies have shown that efficient control of inflammatory disease is associated with a more favorable outcome in patients at risk of developing dementia [13]. At least one study has shown that the use of disease-modifying agents early in a patient’s disease course is correlated with a lower risk of dementia development compared to those patients not efficiently controlling their inflammatory comorbidities [14]. By controlling inflammation, we may hope to limit the morbidity of both rheumatoid arthritis and dementia. However, we know that certain disease-modifying agents should be avoided to prevent contributing further neurological insults [10]. Further research into alternatives to classic treatment options is necessary and ongoing. One promising beacon is the role of inhibitors of proinflammatory cytokines, such as tumor necrosis factor inhibitors [13]. Refinement and alternatives to the current treatment approaches will be inevitable to optimize patient care.

## Conclusions

Frontotemporal dementia is best combated at the prevention stage, and understanding the risk factors is key to treatment and addressing disease progression. This patient’s rheumatoid arthritis history and APS diagnosis were likely not taken into account as serious risk factors for the development of dementia. Although he was treated as accurately as possible based on current guidelines, dementia was not screened for because it has not been emphasized in the clinical sphere. In the years to come, the expanding body of literature will continue to reveal the correlation between inflammatory states and dementia. It is possible that if his rheumatoid arthritis history had been considered a risk factor for dementia, the neuropsychiatric changes taking place could have been recognized sooner. Earlier clinical suspicion along with tracking the progression of the changes may have prompted intervention sooner, most probably improving the patient’s quality of life sooner. After all, improving a patient’s quality of life is the utmost goal of medicine.
